# The potential of the Mediterranean diet to improve metabolic control and body composition in youths with Type 1 Diabetes Mellitus

**DOI:** 10.1186/s12902-024-01593-6

**Published:** 2024-05-09

**Authors:** Didem Güneş Kaya, Neslihan Arslan, Feride Ayyıldız, Elvan Bayramoğlu, Hande Turan, Oya Ercan

**Affiliations:** 1 Istanbul University Cerrahpasa- Cerrahpasa Faculty of Medicine, Pediatrics, İstanbul, Turkey; 2 Faculty of Health Sciences, Department of Nutrition and Dietetics, Erzurum Technical University, Yakutiye, Erzurum, Turkey; 3 Department of Nutrition and Dietetics, Faculty of Health Sciences, Gazi University, Emek, Ankara, Turkey; 4 Istanbul University Cerrahpasa - Cerrahpasa Faculty of Medicine, Pediatric Endocrinology, İstanbul, Turkey

**Keywords:** Mediterranean diet, Type 1 diabetes mellitus, Nutrition

## Abstract

**Background:**

A chronic autoimmune disease with an increasing incidence rate, type 1 diabetes mellitus (T1DM) is typified by the degeneration of the pancreatic beta cells. Diabetes management is significantly impacted by nutrition. Although it has been demonstrated that following the Mediterranean diet (MD) improves metabolic control with type 2 diabetes in children and adults, its effects on children with T1DM have not received much attention.

**Objective:**

Therefore, the purpose of this study was to assess whether adherence to Mediterranean diet is associated with better metabolic control and body composition in youths with Type 1 Diabetes Mellitus. The study recruited T1DM patients aged 6-18 years at İstanbul University Cerrahpaşa Medical Faculty Hospital's Pediatric Endocrinology and Diabetes Outpatient Clinic for follow-up.

**Methods:**

In addition to demographic variables, some anthropometric measurements, body composition and biochemical parameters such as: Trygliceride(TG), Total cholesterol (TC), High density lipoprotein cholesterol (HDL-C), Low density lipoprotein cholesterol (LDL-C), (Aspartate aminotransferase) AST, Alanine transaminase (ALT) and glycated hemoglobin (HbA1c) was analyzed. The time in range (TIR) is a value obtained from continuous glucose monitoring. KIDMED was used to assess the participants' adherence with the MD.

**Results:**

Good adherence to the MD resulted in much larger height SDS than poor adherence. Poor adherence to MD resulted in higher body fat than moderate and good adherence.

There is positivite correlation between TIR and KIDMED score. Adherence to MD is negatively associated with HbA1c. The regression anaylsis showed that a one-point rise in the KIDMED score would result in a 0.314-unit reduction in the HbA1c value (*p* < 0.01).

**Conclusions:**

In conclusion, this study found that adhering to MD led to improved anthropometric measurements, biochemistry, and diabetes outcomes. Awareness among children, adolescents with T1DM, and their parents about the benefits of MD compliance for glycemic and metabolic control should be raised.

## Introduction

Type 1 diabetes mellitus (T1DM) is a chronic autoimmune disease with a growing incidence rate that is characterized by the destruction of the beta cells of the pancreas [1]. According to the International Diabetes Federation's 2022 report, there are 8.75 million individuals diagnosed with T1DM worldwide and an average of 1.52 million of these individuals are children and adolescents under the age of 20 [2]. According to the International Diabetes Federation diabetes atlas, the prevalence of Type 1 DM in the 0-19 age range is stated as 25.8 per 1000 [2]. While insulin therapy is the most vital part of the treatment approach to T1DM, medical nutrition therapy (MNT) also plays an important role in improving the course of the disease and reducing the risk of complications [3, 4]. International guidelines recommend that optimal growth and development, acquiring and maintaining healthy eating habits, and achieving weight control should be the main considerations for MNT for children and adolescents with diabetes. Aiming to prevent acute and chronic complications is also among these recommendations [3, 5]. Complications of T1DM include obesity and cardiovascular diseases [6–8]. Cardiovascular diseases are the primary cause of mortality and morbidity for diabetic patients [9].

Today, many dietary models (such as a low-carbohydrate diet, Mediterranean diet (MD), and intermittent fasting) are practiced by individuals with diabetes [10–12]. Among these diets, the MD is reported to improve glycemic control and reduce the risk of subsequent metabolic diseases of children with T1DM by helping with balanced and adequate nutrition [13]. One study reported that poor adherence to the MD increases the risk of obesity, hypertriglyceridemia and insulin resistance in healthy children [14]. The MD is a plant-based, balanced and sustainable diet model that emphasizes the consumption of fresh fruits and vegetables, legumes, whole grains, nuts, fish and especially olive oil and limits red meat consumption [15]. The Mediterranean diet provides balanced and diverse macronutrients, as well as low saturated fat content, high monosaturated fatty acid content, fiber, complex carbohydrates, and significant antioxidant content[16], preventing many chronic diseases, especially obesity and type 2 diabetes [17]. The Mediterranean Diet Quality Index for Children and Adolescents (KIDMED) is a widely used and practical tool used to assess adherence to the MD in children [18]. The reliability of the Turkish adaptation of KIDMED has been validated and its applicability for the Turkish population has been demonstrated [19]. KIDMED is a simple frequency of consumption questionnaire on indicators of adherence to the MD [20].

Studies on adherence to the MD in children with T1DM are limited in number [20–24]. In a study conducted with young T1DM patients aged 10 years and older, the relationship between inflammation indicators and the KIDMED scores was investigated; however, no significant results were found [22]. Two other studies found an inverse relationship between the KIDMED scores and glycated hemoglobin (HbA1c) levels [20, 21], while another found a negative relationship between the KIDMED scores and the risk of hyperglycemia [23]. In yet another study, significant positive results were obtained regarding cholesterol levels using MD intervention [24]. However, other studies in the literature had different objectives and methods and achieved varying results.

Studies about relationship between KIDMED scores and metabolic control, the lipid profile, and body composition (BC) of children and adolescents with T1DM are limited. Therefore this this study aimed at determining the relationship between adherence to the MD and metabolic control, the lipid profile, and BC of children and adolescents with T1DM.

## Methods

### Study design

This study was conducted on 128 individuals with Type-1 diabetes between the ages of 6-18. Questions regarding demographic data and KIDMED were asked to individuals using the survey method. Anthropometric measurements of the individuals were taken by the researchers using appropriate methods, and their body compositions were evaluated with the bioelectrical impedance method.

### Sample

T1DM patients between the ages of 6 and 18 years who were followed-up in the Pediatric Endocrinology and Diabetes Outpatient Clinic of İstanbul University Cerrahpaşa Medical Faculty Hospital were included in the study. The G*Power program was used to determine the required sample size. According to the results of the power analysis, a sample size of 128 participants was required for an 80% confidence interval (CI) at 5% statistical significance.

Participants were invited to participate in the study during routine outpatient clinic visits for 1 year. The study was conducted in accordance with the Declaration of Helsinki. The participants and their families were provided with detailed information about the study and their informed consent was obtained. Individuals with T1DM aged 6-18 who were followed-up for at least 1 year and were not in the honeymoon phase of the disease were included in the study.

In addition to demographic characteristics such as age and sex, some anthropometric measurements (body weight, height, and waist circumference (WC)), BC, and some biochemical parameters [HbA1c, high-density lipoprotein cholesterol (HDL-C), low-density lipoprotein cholesterol (LDL-C), total cholesterol (TC), triglycerides (TG), aspartate aminotransferase (AST), and alanine transaminase (ALT) values] were evaluated. The participants' adherence to the MD was measured using KIDMED.

### Anthropometric measurements and body composition

Anthropometric measurements of the individuals included in the study (body weight, height, and waist circumference) were taken by a single authorized health personnel. A Tanita MC780 Body Composition (BC) Scale was used for the body weight and BC measurements. Lean mass (kg), lean mass (%), muscle (kg), fluid (kg), fluid (%), fat (kg), fat (%), bone (kg), body fat (kg), and fluid mass (kg) were used to calculate the BC of the participants [25]. A Harpenden stadiometer was used for height measurement [26]. The body mass index (BMI) was calculated using the formula: weight (kg) / height (m^2^). The standard deviation scores (SDS) of the participants were calculated using Neyzi's standardized percentile curves for Turkish children [27].

The standardized percentile curves for Turkish children were used to evaluate the waist circumference percentiles [28]. Fat percentage percentile values, which are recommended for use in assessing mildly obese and obese children in clinical practice, were calculated using percentile curves for Turkish children and adolescents [29]. Those with body fat percentiles below 2 were classified as underfat, between 2 and 85 as normal, between 85 and 94 as overfat, and 95 and above as obese. The visceral adiposity index was calculated using formula for genders: for males: {[WC/39.68 + (1.88 × BMI)] × (TG/1.03) × (1.31/HDL)}; for females: {[WC/36.58 + (1.89 × BMI)] × (TG/0.81) × (1.52/HDL)} [30]. In accordance with WHO guidelines, participants with BMI values of <–3SD were interpreted as severe underweight, <–2SD as underweight, >+1SD as overweight/tall, >+2SD as obese [31].

### Biochemical parameters

HbA1c, HDL-C, LDL-C, TC, TG, AST, and ALT values were analyzed in the biochemistry laboratory of İstanbul University-Cerrahpaşa, Cerrahpaşa Medical Faculty Hospital. The metabolic classification of the participants was classified as good (<7 mg/dL), moderate (7–9 mg/dL), and bad (>9 mg/dL) according to the HbA1c recommendations of the International Society for Pediatric and Adolescent Diabetes [32]. Their lipid profiles were interpreted according to the National Cholesterol Education Program criteria, and the dyslipidemia diagnoses followed the same criteria [33]. The insulin dose of the participants per kilogram of body weight was recorded via the patient files.

The time in range (TIR) is a value derived from continuous glucose monitoring that indicates the percentage of time an individual's blood glucose level stays between 70 and 180 mg/dL. The TIR is a calculation used for evaluating individuals with diabetes on insulin. A value above 70% is classified as good and below 70% is classified as poor [34–36].

### KIDMED

KIDMED was developed by Majem et al. to assess adherence to the MD of children and adolescents [37]. The scale was adapted to Turkish by Kaya et al. [38]. KIDMED consists of 16 questions that are answered as Yes or No. Of these questions, 12 are scored positively and 4 are scored reversely. Yes answers to positively scored questions are scored +1 points, and Yes answers to reverse scored questions are scored +1 points. KIDMED has a score range of 0 to 12. The total KIDMED scores are categorized as follows: poor adherence (total score of 3), moderate adherence (total score between 4 and 7) and good adherence (total score of 8) [38].

## Statistical analysis

All analyses were performed using IBM SPSS Statistics for Windows 24.0 (IBM Corp., Armonk, NY, USA). Descriptive characteristics were presented for all participants of both sexes. After verifying that the continuous variables were normally distributed with the Shapiro-Wilk test and probability-probability (PP) plot, descriptive characteristics were presented as the mean and standard deviation, while qualitative variables were presented as the frequency or median. The chi squared test, t test for independent variables (means), and Mann-Whitney U test (medians) were used to examine differences between the sexes and p-values of all of the tests were calculated. ANOVA was applied to compare multiple groups. Correlation analysis was conducted using the Pearson r (parametric data) or Spearman r (nonparametric data) tests, depending on the characteristics of the associated variables. A linear regression model was used to further examine the relationship between the HbA1c values and age, sex, KIDMED scores, and BMI, and TC, LDL-C, and HDL-C values. Correlation coefficient analysis was conducted to examine the linear relationship between the variables.

Correlations with a r/r or *p*-value of < 0.05 or < 0.01 were considered as statistically significant. The CI was set at 95%. The absolute values of the Pearson and Spearman correlation results were interpreted as very strong for values between 0.9 and 1, strong between 0.7 and 0.89, moderate between 0.40 and 0.69, weak between 0.10 and 0.39, and insignificant between 0 and 0.10 [39]. Linear multiple regression was used to understand the predictive power of the varibles in relation to HbA1c. HbA1c was entered as the dependent variable of the model. The other varibles related to it were entered as independent variables.

## Results

**Fig. 1:**
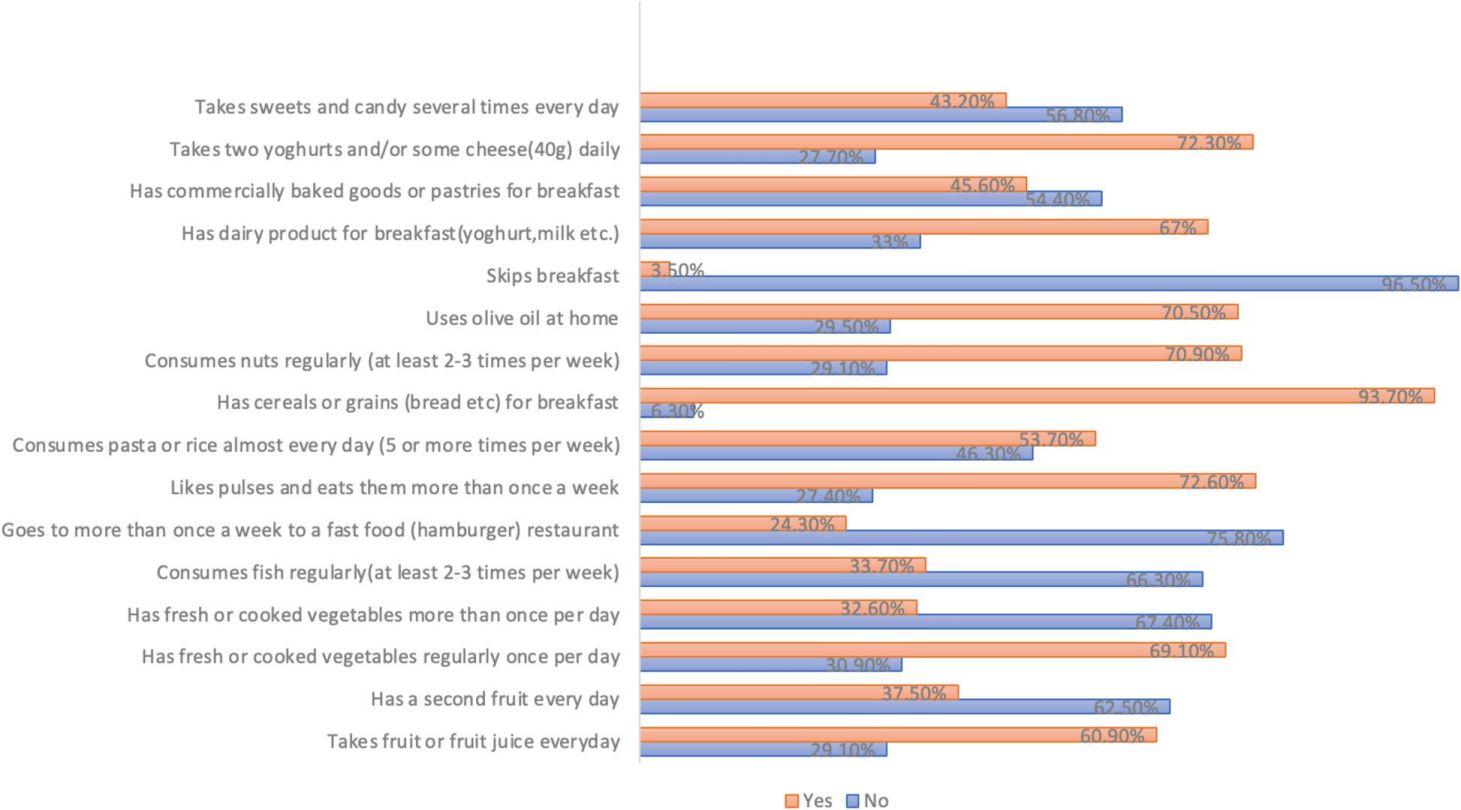
KIDMED Respondents’ answers

The study included 285 children and adolescents (126 females and 159 males), aged 6–18 years, who had been diagnosed with T1DM. Anthropometric measurements, some biochemical parameters, and general characteristics of the individuals according to their KIDMED classifications are shown in Tables 1 and 2. The participants' responses to the KIDMED questions are reported in Figure 1. The mean age of the participants was 13.7 ± 3.63 years, and 27.4% were prepubertal, while 72.6% were pubertal. Among the participants, 84.2% used pen insulin injectors, 15.8% used insulin pumps, and 28.4% (n:81) had a sensor. It was determined that 31.9%, 43.9%, and 24.2% of the participants had poor, moderate, and good metabolic control, respectively. Moreover, 43.5% had dyslipidemia, 4.2% had complications in the form of nephropathy, 8.1% had comorbid hypothyroidism, and 49.4% of individuals with sensors had an appropriate TIR, while 50.6% had a low TIR. The mean KIDMED score of the patients was calculated as 6.42 ± 2.5. According to their KIDMED score categorizations, 17.5% of the participants had poor adherence, 43.2% had moderate adherence, and 39.3% had good adherence to MD.

**Table 1:** Antropometric measurements according to KIDMED classification

	**Total (n:285)**	**Poor (n:50)**	**Moderate (n:123)**	**Good (n:112)**	***p***
**Height (cm)**	156.4±19.45	158.4±16.74	156.7±16.42	155.2±23.33	0.624
**Height (SDS)**	0.2±1.03	-0.1±1.07^a^	0.1±0.97^a,b^	0.4±1.05^b^	<0.01*
**Body weight**	53.1±18.60	58.6±22.93	51.9±14.72	52.1±19.97	0.071
**Body weight (SDS)**	0.3±3.54	0.2 ±1.54	0.1±1.25	0.6±5.41	0.621
**Body mass index (kg/m**^**2**^**)**	21.0±6.01	23.5±11.98^a^	20.6±3.37^b^	20.2±3.61^b^	<0.01*
Obese	%6.7 (n:19)	%14.0 (n:7)	%6.5 (n:8)	%6.7 (n:19)	
Overweight	%15.1 (n:43)	%18.0 (n:9)	%17.1 (n:21)	%15.1 (n:43)	0.176
Normal	%73.7 (n:210)	%64 (n:32)	%70.7 (n:87)	%73.7 (n:210)	
Underweight	%3.9 (n:11)	%4 (n:2)	%4.1 (n:5)	%3.9 (n:11)	
Severely underweight	%0.7 (n:2)	-	%1.6 (n:2)	%0.7 (n:2)	
**Body mass index** (z score)	0.2±3.55	1.4±7.99	0.1±1.36	-0.0±1.18	0.039
**Fat free mass (kg)**	40.0±12.55	43.3±13.86	39.2±10.84	39.5±13.53	0.134
**Muscle mass (kg)**	38.0±11.96	41.0±13.23	37.2±10.34	37.5±12.89	0.136
**Fluid (kg)**	28.6±8.73	31.3±9.85	28.1±7.55	28.0±9.26	0.058
**Fat mass (kg)**	13.0±7.19	15.6±10.29^a^	13.0±6.43^a,b^	11.8±5.94^b^	<0.01*
**Bone mass (kg)**	2.0±0.64	2.2±0.68	2.0±0.56	2.0±0.7	0.307
**Trunk fat mass (kg)**	6.3±4.04	7.9±6.42^a^	6.0±3.08^b^	5.9±3.43^b^	<0.01*
**Trunk fat**	27.1±13.92	29.1±15.03	26.1±12.90	27.4±14.49	0.426
**Fat free mass (%)**	76.2±6.92	75.0±7.80	75.8±7.19	77.2±6.09	0.108
**Fluid percentage**	57.4±38.87	54.4±5.95	57.8±46.99	58.3±37.67	0.831
**Fat percentage (%)**	23.6±6.92	24.9±7.80	24.0±7.10	22.7±6.21	0.130
**Fat percentage(percentile)**	59.7±28.97	65.4±27.95	60.3±27.84	56.5±30.4	0.194
**Classification of fat percentage**					
Normal (223)	%78.2	%68	%80.5	%80.4	0.01*
Overfat (n:40)	%14.0	%14	%10.6	%17.9	
Obese (n:22)	%7.7	%18	%8.9	%1.8	
Visceral adiposity index	1.2±1.00	1.4±1.41	1.2±0.84	1.0±0.91	0.055
Waist circumference	71.5±9.47	74.6±12.63^a^	70.6±8.36^b^	71.1±8.78^a,b^	0.038*
Waist circumference persentil	58.5±32.80	64.6±31.82	56.0±35.07	58.6±30.50	0.294

Categorical variables represented as (%). χ2: Chi squared. Significance set at *p*-value< 0.05

* indicates statistical significance

^a,b^indicate a significant difference according to Anova result

**Table 2:** Biochemical measurements and some descriptive variables according to KIDMED classification

	**Whole group (n:285)**	**Poor (n:50)**	**Moderate (n:123)**	**Good (n:112)**	***p***
**Age (years)**	13.7±3.63	14.1±3.26	13.7±3.45	13.6±3.99	0.734
**Insulin treatment**					
Insulin injection (n:240)	%84.2	%92.0	%91.9	%72.3	<0.01*
Insulin pump (n:45)	%15.8	%8.0	%8.1	%27.7	
**Sensor use**					
Yes (n:81)	%28.4	%12.0	%25.2	%39.3	<0.01*
No (n:204)	%71.6	%88	%74.8	%60.7	
Insulin dose (units/kg)	0.9±0,28	1.1±0.28^a^	0.9±0.27^b^	0.8±0.27^b^	<0.01*
**Metabolic control**					
Good (n:91)	%31.9	%6.0	%17.9	%58.9	<0.01*
Moderate (n:125)	%43.9	%28.0	%58.5	%34.8	
Bad (n:69)	%24.2	%66.0	%23.6	%6.3	
HbA1c	7.9±1.53	9.49±1.56^a^	8.1±1.26^b^	7.1±1.19^c^	<0.01*
Time in range (TIR) (%)	73.9±16.63 (n:81)	56.3±21.26 (n:6^)a^	65.6±17.65 (n:31)^a^	81.5±9.69 (n:44)^b^	<0.01*
**TIR classification**					
> 70%	%49.4 (n:40)	%33.3 (n:2)	%29.0 (n:9)	%65.9 (n:29)	<0.01*
<70%	%50.6 (n:41)	%66.7 (n;4)	%71.0 (n:22)	%34.1 (n:15)	
TC (mg/dL)	159.1±37.21	168.4±35.37^a^	162.1±37.15^a,b^	151.7±37.00^b^	<0.01*
LDL- C (mg/dL)	93.6±27.62	94.3±26.82	97.4±26.62	89.4±28.69	0.093
HDL- C (mg/dL)	60.9±17.79	62.8±24.40	59.6±16.49	61.4±15.63	0.508
TG (mg/dL)	103.0±56.23	123.1±70.4^a^	104.9±50.2^a,b^	91.9±53.16^b^	<0.01*
AST (U/L)	18.0±8.11	18.5±7.24	17.7±8.49	18.2±8.10	0.810
ALT (U/L)	15.4±8.46	18.2±12.77^a^	14.2±7.37^b^	15.5±6.82^a, b^	<0.01*
Complications					
Yes (n:12)	%4.2	%8.0	%4.9	%1.8	0.170
No (n:273)	%95.8	%92.0	%95.1	%98.2	
**Comorbidity**					
Yes (n:23)	%8.1	%6.0	%7.3	%9.8	0.655
No (n:262)	%91.9	%94.0	%92.7	%90.2	
**Dyslipidemia**					
Yes (n:124)	%43.5	%56.0	%52.0	%28.6	0.000*
No (n:161)	%56.5	%44.0	%48.0	%71.4	
**Pubertal situation**					
Prepubertal (n:78)	%27.4	%20.0	%27.6	%30.4	0.392
Pubertal (n:207)	%72.6	%80.0	%72.4	%69.6	

Categorical variables represented as (%). χ2: Chi squared. Significance set at *p*-value< 0.05

*TC* Total cholesterol, *LDL-C* LDL- cholesterol, *HDL-C* HDL- cholesterol, *AST* Aspartate aminotransferase, *ALT* Alanine transaminase, *TG* Triglyceride

* indicates statistical significance

^a,b,c^indicate a significant difference according to Anova result

The mean BMI of the participants with poor adherence to the MD was significantly higher than those with both moderate and good adherence (*p* < 0.01). When the BMI SDS values of the participants were analyzed, it was observed that 73.7% had a normal BMI. The percentages of obese, overweight, underweight, and extremely underweight individuals were 6.7%, 15.1%, 3.9% and 0.7%, respectively. There was no correlation between the KIDMED classification and BMI.

It was observed that the fat mass (kg) and waist circumference of those with poor adherence to the MD were higher than those with good adherence (*p*<0.01 and *p* = 0.038, respectively). Participants with poor adherence to the MD had significantly higher body fat than those with both moderate and good adherence (*p* < 0.01). When the body fat percentile groups of the patients were examined, it was observed that the moderate and good adherence groups were composed of significantly more non-overweight/non-obese individuals, while only the good adherence group had a significantly lower ratio of obese individuals (*p* = 0.01).

While 65.9% of the participants with good adherence had a TIR above 70%, 29.0% of those with moderate adherence and 33.3% of those with poor adherence had a TIR above 70%. The HbA1c and insulin dose (units/kg) were significantly higher in the poor adherence group compared to moderate and good adherence groups (*p* < 0.01). The TC and TG levels of the poor adherence group were higher compared to the good adherence group (*p* < 0.01). The ALT levels of the poor adherence group were only higher than the moderate adherence group (*p*<0.01).

There were significant intergroup differences regarding the type of insulin therapy, presence of sensors, presence of dyslipidemia, and metabolic control between the MD adherence groups. The presence of dyslipidemia was significantly lower in the good adherence group (*p* = 0.000). It was found that individuals with poor adherence were more likely to have poor metabolic control, those with good adherence were more likely to have good metabolic control, and those with moderate adherence were more likely to have moderate metabolic control (*p* < 0.01).

Table 3 shows the correlation coefficients between the KIDMED scores, HbA1c, TIR, and some other variables. Accordingly, it was found that there was a significant negative correlation between the KIDMED scores and the BMI, body fat mass (kg), insulin (units/kg), HBA1C, TC, LDL-C, HDL-C, and TG values and a significant positive correlation was found between the KIDMED scores and TIR (*p < 0.05 and **p < 0.01).

**Table 3:**
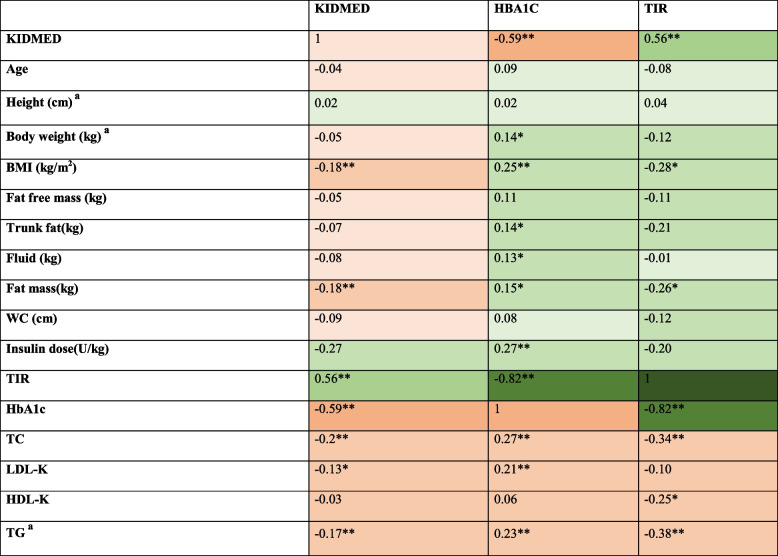
Correlation between KIDMED scores, HBA1C, TIR and some variables

*BMI* Body mass index, *WC* Waist Circumference, *HbA1c* Glycolisided hemoglobin, *TC* Total Cholesterol, *LDL-K* Low density lipoprotein cholesterol, *HDL-K* High density lipoprotein cholesterol, *TG* Triglyceride

^a^Spearman correlation Others: Pearson correlation, *p<0.05 and **p<0.01

Correlation Coefficient Ranges

There was also a significant negative correlation between the HbA1c and KIDMED scores and HbA1c and TIR. There is also a significant positive correlation between the HBA1c value and body weight, BMI, body fat mass, fluid mass, and fat mass, and TC, LDL-C and TG values. The TIR had a significant positive correlation with the KIDMED scores and HBA1c value, and a significant negative correlation with the BMI and fat mass, and TC, HDL-C, and TG values (*p < 0.05 and **p < 0.01).

The analysis results regarding the relationship between the HbA1c value and age, sex, BMI, KIDMED scores, and TC, LDL-C, and HDL-C values are presented in Table 4. A 1-point increase in the KIDMED score decreased the HbA1c value by 0.314 units (*p* < 0.01), a 1-point increase in the BMI increased the HbA1c value by 0.031 units (*p* = 0.012), and a 1-unit increase in the TG value increased the HbA1c value by 0.004 units (*p* = 0.012).

**Table 4:** Regression anaylsis of HbA1c and some other variables

		B	SE	Sig.
Model	Constant	7.880	0.608	<.001
	Age	.016	0.20	.407
	Gender	-.196	0.146	.171
	BMI	**.031**	**0.012**	**.012**
	KIDMED	**-.314**	**0.029**	**<.001**
	TC	.001	0.003	.665
	TG	**.004**	**0.001**	**.012**
	HDL-C	.007	0.005	.148
	LDL-C	.005	0.004	.182

*BMI* Body mass index, *HbA1c* Glycolisided hemoglobin, *TC* Total Cholesterol, *TG* Triglyceride, *LDL-C* Low density lipoprotein cholesterol, *HDL-C* High density lipoprotein cholesterol

## Discussion

The aim of this study was to evaluate the relationship between adherence to the MD and glycemic and metabolic control of children and adolescents with T1DM. To the best of our knowledge, this is the first study in Türkiye that aimed to examine the relationship between adherence to the MD and glycemic and metabolic control of children and adolescents with T1DM. The study included 285 children and adolescents (126 females and 159 males), aged 6–18 years, who had been diagnosed with T1DM.

The main findings of this study can be listed as follows. The study showed that people who adhered well to MD had higher TIR values which refers to percentage of time spent within a target blood glucose range, typically above 70%. Spesifically 65.9% of those with good adherence to the MD had a TIR above 70%. In contrast only 29.0% of those with moderate adherence and 33.3% of those with poor adherence had a TIR above 70%. This suggests a positive association between adherence to the MD and glycemic control, as reflected by TIR. This study also analyzed various biochemical parameters among participants with different levels of adherence to the MD. Participants with poor adherence to the MD exhibited higher levels of several biochemical markers, including HbA1c (a marker of long-term glucose control), insulin (units/kg), TC), triglycerides TG, and ALT. There was an important negative connection between KIDMED and BMI, TC, TG, and TIR. For each 1-point increase in the KIDMED score, there was a significant decrease in HbA1c levels by 0.314 units (*p* < 0.01). This suggests that better adherence to the MD was associated with lower HbA1c levels. Conversely, 1-point increase in BMI was associated with a smaller but still significant increase in HbA1c levels by 0.031 units (*p* = 0.012). Similarly, a 1-unit increase in TG levels was associated with a slight increase in HbA1c by 0.004 units (*p* = 0.012). These findings indicate that adherence to the MD had a more pronounced effect on HbA1c levels compared to BMI and TG levels, suggesting that dietary habits play a significant role in glycemic control.

The American Diabetes Association (ADA) highlighted that nutrition plays a very important role managing diabetes and recommends that an individualized MNT should be provided to individuals with diabetes by practicing dietitians. It was also reported that meal planning and carbohydrate counting in combination with insulin therapy training can improve the glycemic control of individuals with T1DM [40]. The ADA and American Heart Association reported in their scientific statement that the MD pattern improves glycemic control (Level of Evidence B) [41]. One study showed that a MD intervention reduced the HbA1c level from 7.1% to 6.8% in individuals with diabetes [42]. The whole grain content, high fruit and vegetable content, and high antioxidant content of the MD have a positive effect on blood glucose, the HbA1c level, and lipid profile, which is a risk factor for diabetes and affects comorbidities [43]. The potential protective effects of the MD pattern are thought to be due to the effects of the food groups involved in this pattern that reduce oxidative stress, inflammation, and insulin resistance [44]. In one study conducted in Spain, the participants had a mean KIDMED score of 7.04 [20]. When the previous studies conducted in Türkiye were analyzed, it was observed that the mean KIDMED score was reported as 6.4 ± 2.37 in a study including individuals belonging to a similar population to the present study, aged 6–18 years [45]. The present study obtained results similar to those of other studies in the literature (mean: 6.42 ± 2.5). The researches of the aforementioned Spanish study explained their better results with the fact that the study was conducted in a coastal area with high accessibility to olive oil, fresh fruits, vegetables, and fish, and a cultural characterized with high legume consumption, as well as a generally high adherence to the MD [20]. Since the participants of the present study were individuals with T1DM, it is known that they and their parents received training on insulin use and carbohydrate counting. Therefore, it is thought that the participants may have acquired healthy eating habits and their parents, who are responsible for food preparation and cooking at home in Turkish culture, may have acquired methods for healthy meal preparation and healthy cooking. In addition, when the answers given to the questions of the KIDMED were analyzed, it was seen that almost all of the participants did not skip breakfast. It was also seen that about 70% used olive oil at home, and about 60% consumed fruits or vegetables at least once a day. This explains why the mean KIDMED score in this study was 6.42 ± 2.5, close to the upper limit of the middle range [3–6].

In a study conducted by Zhong et al. with individuals with T1DM, the LDL-C and TC values were lower in those with good adherence to the MD [21]. Zhong et al. [21] and Barmpa et al. [46] found no significant difference between the HbA1c values of different MD adherence groups, whereas Dominguez et al. [20] reported that those with good adherence to the MD had lower HbA1c values. In the present study, the HbA1c, TC and TG values were significantly higher in individuals with poor adherence to the MD. In addition, there was a statistically significant negative correlation between the KIDMED scores and the HbA1c, LDL-C, HDL-C, and TC values. Similarly, Dominguez et al. [20] found an inverse relationship between the KIDMED scores and the LDL-C value. Zhong et al. reported that, according to their regression analysis results, a 2-point increase in the KIDMED score decreased the HbA1c value of an individual from 7.5% to 7.43% [21]. In the current study, it was observed that a 1-point increase in the KIDMED score decreased the HbA1c value by 0.314 units, while a 1-point increase in the BMI increased the HbA1c value by 0.004 units. Although the results of some studies vary, these results are in line with the lipid-lowering and glycemic control effect of the MD, which has been demonstrated by longitudinal studies and meta-analyses [21, 47].

The MD helps to both lower lipid levels due to its fiber content and prevent lipids from oxidizing and accumulating in blood vessels due to its antioxidant content [16]. It is known that the fruit and vegetable components of the MD contain high amounts phytosterols, carotenoids, phenolic compounds, and flavanols [48], which lower HbA1c and lipids [42, 49], and improve endothelial function [50, 51]. Evidence suggests that polyphenols increase glucose uptake in muscles, decrease gluconeogenesis in the liver, and inhibit inflammation-induced destruction of pancreatic beta cells [52]. In addition to the effects of its components, the whole of the MD has synergistical and potent antiinflammatory and antioxidative effects [16, 53, 54]. It was reported that the MD may have a protective effect against oxidative stress, which is defined as the imbalance between free radicals and the antioxidant defense system and is one of the most important causes of pancreatic beta cell destruction through metabolic pathways and signaling mechanisms [55]. It is believed that the MD decreases the levels of parameters that increase reactive oxygen species associated with this process and increases antioxidant capacity [55, 56], which may explain the results of the current study. Thus, it is of pivotal importance to recommend the MD pattern to individuals with T1DM during their education. A longitudinal study conducted in Iran showed that the MD reduces the risk of cardiovascular disease and microvascular complications in individuals with T1DM [57]. Consuming a diet consistent with the MD pattern will reduce the risk of comorbid diseases and mortality in individuals with T1DM.

In this study, the number of individuals using sensors was 81 (28.4%). Only these patients had recorded TIR values. The TIR is an important parameter for individuals with T1DM and its clinical and practical use has been increasing in recent years [35, 36]. In 2 studies conducted with children and adolescents with T1DM, a significant positive correlation was found between the KIDMED scores and the TIR [20, 23]. Similarly, the present study also found a positive correlation between adherence to the MD and the TIR in individuals with T1DM. One study investigating the effect of MD intervention on glycemic control in adolescents with T1DM reported that intervention increased the TIR from 52% to 62% [58]. However, the results reported in the literature vary. In an interventional study investigating body weight loss and outcomes related to different dietary patterns in 19–30-year-old individuals with T1DM, Igudesman et al. observed that the MD decreased the TIR, although not significantly. They also reported that the MD increased the time above range (glucose levels below 250 mg/dL) [59]. It is recommended to investigate whether the MD without energy restriction induces hyperglycemia. The association between the TIR and KIDMED reported in the study of Domingez et al. supports the benefit of adherence to the MD on glycemic control [20]. In addition, in the present study, conducted with individuals with T1DM, a significant negative correlation was found between the BMI, TC, and TG and TIR (Table 3). It is thought that the increase in sensor usage will have a positive effect on metabolic parameters.

## Limitations and strengths

When databases were searched, no other study in Türkiye investigating the relationship between the MD and TIR, and HbA1c, HDL-C, LDL-C, TC, and TG values as metabolic control markers of children and adolescents with T1DM was found. This study was completed with 285 participants (126 females and 159 males). The homogeneous sex distribution of the participants was one of the strengths of this study. The TIR values, which have been frequently recommended for clinical use in recent years, of participants using sensors were obtained and used in this study. Since sensors are not reimbursed by health insurance in Türkiye and they are expensive, not every individual has access to them [60]. Thus, the TIR values of all of the participants could not be obtained. In future studies, it would be useful to obtain food consumption records and evaluate them by establishing a relationship with metabolic parameters.

## Conclusion

In conclusion, this study found that individuals with good adherence to the MD exhibited better anthropometric measurements, biochemical parameters, and diabetes-related outcomes. MD education along with insulin therapy, as recommended by the guidelines, to individuals with T1DM will contribute to improving the prognosis of diabetes. In addition to standard glycemic and metabolic control markers, the TIR marker obtained using sensors is very useful for both individuals with T1DM and their parents and researchers [60, 61]. Increasing adherence to MD may improve TIR values. More randomized controlled studies are needed to examine the association of the TIR marker with the MD in children and adolescents with T1DM.

## Data Availability

The datasets used and/or analysed during the current study are available from the corresponding author on reasonable request.
